# Assessing Clinical Features of Adolescents Suffering from Depression Who Engage in Non-Suicidal Self-Injury

**DOI:** 10.3390/children9020201

**Published:** 2022-02-04

**Authors:** Maria Serra, Anna Presicci, Luigi Quaranta, Elvita Caputo, Mariaclara Achille, Francesco Margari, Federica Croce, Lucia Marzulli, Lucia Margari

**Affiliations:** 1 Department of Pharmacy-Pharmaceutical Sciences, University of Bari “Aldo Moro”, Via Edoardo Orabona 4, 70125 Bari, Italy; 2 Department of Neuroscience, Sense Organs and Locomotor System, University Hospital “Policlinico”, Piazza Giulio Cesare 11, 70124 Bari, Italy; presicci.anna@gmail.com; 3 Department of Computer Science, University of Bari “Aldo Moro”, Via Edoardo Orabona 4, 70125 Bari, Italy; quarantalgu@gmail.com; 4 Department of Biomedical Sciences and Human Oncology, University of Bari “Aldo Moro”, Piazza Giulio Cesare 11, 70124 Bari, Italy; elvitacaputo@gmail.com (E.C.); mariaclarachille@gmail.com (M.A.); lucia.margari@uniba.it (L.M.); 5 Department of Basic Medical Science, Neuroscience and Sensory Organs, University of Bari “Aldo Moro”, Piazza Giulio Cesare 11, 70124 Bari, Italy; francesco.margari@uniba.it (F.M.); federicacroce1990@gmail.com (F.C.); lucia.marzulli@uniba.it (L.M.)

**Keywords:** depression, non-suicidal self-injury, self-harm, specifier, adolescent, emotional dysregulation, culture

## Abstract

Depressive disorders (DDs) and non-suicidal self-injury (NSSI) are important juvenile mental health issues, showing alarming increasing rates. They frequently co-occur, mainly among adolescents, increasing the suicide risk. We aimed to compare the clinical features of two groups of adolescents with DDs, differed by their engagement or not in NSSI (“DD + NSSI” and “DD”). We hypothesized that NSSI would characterize particularly severe forms of DDs suitable for becoming specific phenotypes of adolescent depression. We enrolled 56 adolescents (11–17 years) diagnosed with a DD according to the DSM-5 criteria. They were assessed for NSSI endorsement (Ottawa Self-Injury Inventory), depressive symptoms (Children’s Depression Inventory 2), emotional dysregulation (Difficulties in Emotional Regulation Scale), and anxiety symptoms (Screen for Child Anxiety-Related Emotional Disorders). The two groups accounted for 31 (“DD + NSSI”) and 25 (“DD”) individuals. The “DD + NSSI” group had significantly higher suicidal ideation (*p* 0.0039), emotional dysregulation (*p* 0.0092), depressive symptoms (*p* 0.0138), and anxiety symptoms (*p* 0.0153) than the “DD” group. NSSI seemed to characterize more severe phenotypes of adolescent depression, applying for a potential role as a “specifier” of DDs, describing relevant information for their management. Further studies are needed to support this hypothesis and its potential opportunities for prevention and treatment.

## 1. Introduction

Depression is estimated to be one of the main causes of overall disease social and economic burden worldwide [1]. It is a clinical entity well defined by the diagnostic systems in use [2,3], subjected to numerous international guidelines. Depressive disorders (DDs) are characterized by depressed mood, loss of interest or pleasure, and a variety of cognitive and somatic modifications, with a negative impact on the individual functioning [2]. Depression is considered one of the most alarming “new morbidities” among adolescent mental health disorders [4]. According to the most recent National Institute for Mental Health data, 15.7% of American people aged 12–17 years had at least one major depressive episode in 2019 [5]. A very consistent finding across the studies on depression is the female preponderance, with the greatest increase in sex-related incidence occurring in late adolescence [6].

In clinical practice, self-harm behaviors are a rapidly growing mental health problem. The fifth edition of the Diagnostic and Statistical Manual of Mental Disorders (DSM-5) introduced a clinical entity of yet uncertain definition, non-suicidal self-injury (NSSI), as a self-existing diagnostic category, not solely as a symptom of borderline personality disorder (BPD), including it in the third section as a syndrome requiring further study [7]. NSSI involves deliberate and intentional injury to body tissues without intention to die. Prevalence data of NSSI are highly variable in the literature. A systematic review estimated an international pooled prevalence of 17.2% among adolescents in the community [8]. Data from psychiatric inpatients show higher prevalence rates [9]. NSSI can be considered a transdiagnostic phenomenon, observed in many psychiatric disorders such as substance use, BPD, depression, anxiety, and eating disorders [10,11]. In particular, NSSI is highly prevalent in patients with mood disorders, especially if early-onset, and is considered a predictor for suicidal ideation (SI) and behavior (SB) [12,13,14,15,16]. Recent reports from the Center for Disease Control show an escalation in rates of SI and SB among school and preschool-aged children [17,18,19]. The association between DDs and NSSI may increase the suicide risk [15,20].

Our study aimed to compare two groups of adolescents suffering from depression, differentiated by the involvement or not of the NSSI, evaluating their depressive and anxious symptoms, emotional dysregulation, and suicidal ideation.

We hypothesized that the presence of NSSI would characterize particularly severe forms of DDs suitable for becoming specific phenotypes of adolescent depression.

## 2. Materials and Methods

### 2.1. Design and Participants

We recruited adolescents from 11 to 17 years, hospitalized in the Operative Units of Child Neuropsychiatry and Psychiatry of the University Hospital of Bari, Italy, in the years 2020–2021 and diagnosed with a DD according to the DSM-5 criteria.

The study protocol was approved by the Independent Ethical Committee of Azienda Ospedaliero-Universitaria Consorziale Policlinico di Bari and conducted according to the recommendations of the Helsinki Declaration. The parents of all participants provided their written informed consent.

### 2.2. Measurements

All participants underwent the administration of standardized protocols for the assessment of NSSI endorsement, depressive symptoms, emotional dysregulation, suicidal ideation, and anxiety symptoms.

#### 2.2.1. Non-Suicidal Self-Injury

We used an adapted Italian version of the Ottawa Self-Injury Inventory (OSI) to assess the endorsement of self-harm in the recruited patients and to divide the sample into two subgroups: “DD + NSSI” and “DD” [21]. For this purpose, we considered only the following items:

- n. 1 (“How often in the past one month did you actually injure yourself without the intention to kill yourself?”) with the scores from 0 (“Not at all”) to 3 (“Every day”);
- n. 2 (“How often in the past six months did you actually injure yourself without the intention to kill yourself?”) with the scores from 0 (“Not at all”) to 4 (“Every day”).

Among patients with a score ≥ 1 in one or both items (at least one episode of self-injury), we selected those with at least five episodes of NSSI in the past year for the group “DD + NSSI”, as per DSM-5 proposed NSSI criteria [7]. Patients not endorsing NSSI or with less than 5 episodes of self-injury were put in the group “DD”.

#### 2.2.2. Depressive Symptoms

We used the Italian version of the Children’s Depression Inventory 2 (CDI 2): the 28-item self-report version (CDI 2-SR) and the 17-item parent version (CDI 2-PV) [22]. This questionnaire is commonly used in pediatrics (7–17 years) to provide information about the presence and severity of depressive symptoms in the past two weeks. Each item is rated on a 3-point Likert-type scale from 0 to 2 for the CDI 2-SR and on a 4-point Likert-type scale from 0 to 3 for the CDI 2-PV. High internal consistency values were demonstrated in a clinical sample for the total score of the CDI 2-SR (alpha 0.87) and the CDI 2-PV (alpha 0.89) [22]. We used the total scores of both the scales and the four subscale items provided by the CDI 2-SR, measuring symptoms of (1) depressed mood/somatic symptoms; (2) negative self-esteem; (3) ineffectiveness; (4) interpersonal problems. We used T-scores normed based on age and sex [22]. T-scores ≥ 65 were considered positive for depressive symptoms, as per the CDI 2 scoring guidelines for clinical samples [22].

#### 2.2.3. Emotional Dysregulation Symptoms

We used the 36-item Italian version of the Difficulties in Emotion Regulation Scale (DERS) to measure difficulties in identifying and regulating negative emotions [23,24]. Each item is rated on a 5-point Likert-type scale from 0 (“Almost never”) to 4 (“Almost always”). DERS is considered a valid and reliable measure of emotion dysregulation in adolescents (alpha 0.93) [25]. A high internal consistency (alpha 0.90) of the Italian adaptation was demonstrated [24]. We used the total score, which ranges from 36 to 180, with higher scores indicating greater difficulties regulating emotions (no official clinical cut-offs have been identified so far).

#### 2.2.4. Suicidal Ideation

We assessed the presence of suicidal ideation by selecting item 8 of the CDI 2-SR as a dichotomous variable: score 0 (“I do not think about ending my life”), absent; score 1 (“I think about ending my life, but I would never do it”) or 2 (“I want to end my life”), present.

#### 2.2.5. Anxiety Symptoms

We used an adapted Italian version of the 41-item Screen for Child Anxiety-Related Emotional Disorders (SCARED), a self-report (SCARED-SR), and a parent version (SCARED-PV) instrument commonly used to identify anxiety in 9- to 18-year-old individuals [26,27]. Each item is rated on a 3-point Likert-type scale from 0 (“Not true”) to 2 (“Very true”). Suitable internal consistency (alpha 0.90) and discriminant validity were demonstrated for both the scales [26]. Large correlations between parent and child versions were demonstrated [28]. We used the total score of both the scales, which ranges from 0 to 82. Cut-off scores have not been ascertained, although a total score ≥ 25 has been suggested as indicating the presence of an anxiety disorder (more specific if ≥ 30) [27].

### 2.3. Statistical Analysis

We used descriptive statistics and frequency analysis to analyze demographic and clinical data. To evaluate between-group differences in continuous variables, we first verified the *t*-test assumptions of normality and homogeneity of variance through the Shapiro–Wilk test and Bartlett’s test, respectively. Then, based on the results, we distinguished three cases: (1) if both assumptions were met, we performed a two-sided Student’s *t*-test; (2) if the normality assumption was met, but the groups had inhomogeneous variances, then we used a two-sided Welch’s *t*-test; (3) if the normality assumption was violated, we resorted to a nonparametric test, the two-sided Mann–Whitney U-test. When a statistical significance was met using the *t*-test, but a large effect size was not reached (Cohen’s d < 0.8), we used a nonparametric M-W-U-test. For the statistical comparison of categorical variables, we used the one-sided Fisher exact test.

The main tools used for data analysis were “Pandas” and “SciPy”, two scientific computing libraries written for the Python programming language. The threshold of statistical significance was set at *p* < 0.05.

## 3. Results

### 3.1. Demographic and Clinical Data

The sample included 56 individuals, 39 females (69.6%) and 17 males (30.1%), from 11 to 17 years (mean age: 14.5 ± 1.8 years). The participants were all diagnosed with a DD, according to DSM-5 criteria [2]. The “DD + NSSI” group accounted for 31 individuals (55.4%; F:M ratio 5:1), while the “DD” group accounted for 25 patients (44.6%; F:M ratio 1:1). At the recruitment, 18 patients were being treated with individual psychotherapy. Thirty-eight were referred to cognitive-behavioral psychotherapy or relational family therapy at the end of the diagnostic process. Thirty-seven patients received pharmacotherapy. Nine of them had a previous history of psychotropic drugs use, while the others started the treatment during the recruitment process. All the patients underwent the assessment protocol. SCARED-PV and DERS data were not obtained on one and two participants, respectively, due to incomplete compilations. Complete descriptive statistics of demographic and clinical data are summarized in Table 1.

### 3.2. Between-Group Differences Based on Sex

The comparisons between the scores of CDI 2, DERS, and SCARED based on sex are reported in Table 2. Females reported higher depressive symptoms than males for the total score and the four subscale items of the CDI 2-SR, but the difference was statistically significant only for the subscale Negative Self-Esteem (*p* 0.0279). The between-group difference was not statistically significant if depressive symptoms were reported by parents (CDI 2-PV). No significant differences were found when comparing the DERS total scores in the two groups. Females reported suicidal ideation more frequently than males (*p* 0.0163). Anxiety symptoms were significantly higher in the female group at the self-report version of the SCARED (*p* 0.0001) but not at the parent version.

### 3.3. Between-Group Differences Based on NSSI Endorsement

The comparisons between CDI 2, SCARED, and DERS scores in the two groups of patients, split on NSSI endorsement, are reported in Table 3. The “DD + NSSI” group showed significantly higher depressive symptoms at the CDI 2-SR, total score, than depressed patients without NSSI (*p* 0.0138). In particular, they were significantly more impacted than non-self-injuring individuals in the subscales of negative self-esteem (*p* 0.0002) and interpersonal problems (*p* 0.0387). The between-group difference at the CDI 2-PV was not statistically significant. Furthermore, significantly higher emotional dysregulation symptoms were reported by self-injuring depressed patients compared with patients not endorsing NSSI (*p* 0.0092). Self-injuring adolescents reported suicidal ideation more frequently than non-self-injuring ones (*p* 0.0039). Anxiety symptoms were significantly higher in the self-injuring group of depressed patients at the self-report version of the SCARED (*p* 0.0153) but not at the parent version.

## 4. Discussion

This study aimed to identify clinical differences among adolescents suffering from depression based on engagement or not in NSSI.

The relevance of our study is motivated by the increasing prevalence of DDs and NSSI among adolescents in the last few years, with a dramatic worsening during the COVID-19 emergency [29,30]. In a large longitudinal cohort of 1241 Chinese students between 9 and 16 years, depressive symptoms and NSSI increased from 18.5% to 24.9% and from 31.8% to 42.0%, respectively, from before to after lockdown [31].

The female preponderance is one of the most consistent findings across studies about major depressive disorder (MDD), maybe due to biological susceptibility, lower baseline self-esteem, and a higher likelihood of exposure to stress related to interpersonal violence and gender inequity [6,30]. The present sample is aligned on this evidence, with a female percentage of 69.6% of all the recruited subjects. Notably, in our sample, NSSI was five times more frequent in girls than boys. Evidence of more frequent depression and NSSI engagement in females than males is ascertained by the literature [32,33,34]. In girls, and not in males, a bidirectional relationship over time between depression and self-harm was demonstrated, with the one predictive of the other in a dynamic system of psychopathological processes [33].

Among the CDI 2-SR depressive subscales, the negative self-esteem was significantly higher in females than males. Negative self-image and low self-esteem were found particularly associated with NSSI in girls [32,33]. It looks like that in women, depression and self-harm may easily enter in a self-generating vicious cycle where some symptoms (i.e., negative self-image) and not others are associated with significantly higher levels of self-harm [33].

In this sample, females reported SI more frequently than males. This finding could reflect what the literature reports as “the gender paradox of SB”, with females making more suicide attempts than males and men being four to five times more likely to die in their attempts than women [35]. Moreover, MDD is thought to account for a higher incidence of SB in females [35].

In this study, females were significantly more anxious than males. The literature reports higher rates of anxious-depressive comorbidity among community samples of girls, particularly in adolescence [36,37].

In our sample of 56 adolescents suffering from depression, more than half endorsed NSSI. This association is supported by studies of the last decade. Self-harm is commonly assumed as a dysfunctional way of regulating or expressing high emotional distress such as depression, anxiety, or self-hate [33]. Since depression manifests as a combination of sadness, worthlessness, guilt, and other negative feelings, it may function as a precipitating factor of NSSI [38]. Depressive feelings were recognized as risk factors for the development of self-harm a few years later in adolescents [39,40].

In our sample, patients who self-injured reported a significantly higher rate of SI than non-self-injuring ones. Furthermore, they were significantly more emotionally dysregulated, depressed, and anxious than individuals not endorsing NSSI. These results were partly expectable considering the recent literature.

In this sample, patients suffering from depression who engaged in NSSI reported significantly higher rates of SI than non-self-injuring ones. NSSI frequently coexists with suicidal behavior, but their relation is not univocal [41]. On the one hand, some authors considered NSSI as a compromise to avoid total destruction (“anti-suicide model”), with several acts of microsuicide creating an illusion of control of death [41]. According to this theory, NSSI would serve the function of self-regulation, useful to prevent suicide. On the other hand, several studies suggested NSSI as a significant predictor of later suicide attempts [9,42]. In this sight, self-injuring events may increase the likelihood of suicide attempts by developing a sort of habituation or acquired capability to self-injure [43,44,45]. The risk of death by suicide is considered higher during the first six months after an NSSI episode [41].

In our study, significantly higher levels of emotional dysregulation symptoms were reported by the “DD + NSSI” group compared with the “DD” group. Adolescents are particularly vulnerable to NSSI, maybe due to their emotional reactivity and impulsiveness induced by brain maturation [10]. Affective regulation is reported as the most endorsed NSSI function, serving to escape a negative state or to induce a positive one so that people engage in NSSI to regulate stressful emotions in the absence of better strategies of control [38]. Several models of NSSI based on emotional dysregulation were developed, stating that self-harm is induced by a high frequency of negative emotional states and a low tolerance of these feelings [10,43]. In this conceptualization, difficulties in awareness, understanding, and acceptance of emotions as well as in inhibition of impulsive behaviors when facing emotional distress can lead to NSSI engagement as an emotion-regulation strategy [38]. In a recent study of an adult community sample, greater emotional dysregulation was found in self-injurers compared with individuals without a history of self-harm [38]. Our findings confirm the idea that high levels of emotional dysregulation are a common finding in people who self-injure and particularly in a sample of adolescents suffering from depression. On the other hand, depression is itself a syndrome of stress and emotion dysregulation. The DERS, in a study in support of the measure’s construct validity, showed strong correlations with psychological problems, including depression [25]. A high dysregulation profile was predictive for later depression in a sample of German children [46].

Patients endorsing NSSI reported significantly higher levels of depressive symptoms, particularly those related to negative self-esteem and interpersonal problems, than patients without NSSI. In children and adolescents hospitalized for SI or SB, more severe depressive symptoms were one of the factors associated with NSSI [47]. Similarly, in a community sample of adults, current self-injurers had higher depressive levels than individuals without a history of NSSI and subjects who had given up self-injury [38]. In a study of depressed children, those with NSSI were more irritable and with higher externalizing behaviors and depression severity than those without NSSI and SI or SB [19]. In particular, among depressive symptoms, negative self-image and low self-esteem were reported frequently in association with NSSI [32,33]. Concerning the significantly higher levels of interpersonal difficulties reported by the group “DD + NSSI”, longitudinal data showed low mood and insecure peer relationships, increasing the likelihood of self-harming behaviors in young adolescents [48]. Moreover, interpersonal problems are frequently observed in people who self-injure, given that the resolution of an interpersonal problem is one of the expectations preceding the engagement in self-injurious behavior, according to the DSM-5 proposed criterion B of NSSI [7]. The interpersonal function is considered one of the most important endorsed in NSSI: it encompasses attempts to communicate distress, to have interpersonal influence, and to actively hurt or punish others [43].

Anxious symptoms were significantly higher in the “DD + NSSI” group at the self-report version of the SCARED. Anxiety disorders are one of the main co-occurring disorders when talking about NSSI. People with high anxiety were one of the NSSI subgroups found by Klonsky and Olino [49], suggesting that high anxiety symptoms are a common finding in self-injurers. Anxiety disorders were identified as risk factors for NSSI in several longitudinal studies [50].

From our findings, it looks like a group of adolescents affected by depression could be clinically differentiated by others based on NSSI endorsement, which could contribute to the psychopathology of the DD. In our study, the presence of NSSI is likely to define a subgroup of adolescents suffering from depression with specific features, clinically more severe, suitable for specific treatment options. These findings could contribute to the knowledge of the condition NSSI, now considered in more than one way: an isolated condition common in the general population, a transdiagnostic symptom, and a potential self-existing diagnostic category. When trying to conceptualize and better understand the NSSI phenomenon, we need to consider the cultural background where it manifests. What emerges from the recent literature is that NSSI is a global phenomenon, occurring both in western and non-western cultures [51]. Its prevalence rates are increasing globally, including developing countries such as Uganda or Jamaica, with very recent estimates reaching 25.5% for boys and 32.6% for girls [52,53]. Many aspects are similar across different cultures (e.g., prevalence, age of onset, and self-injuring methods), while others are quite different. For example, NSSI behaves as a maladaptive coping mechanism to regulate preferentially intrapersonal issues in western cultures and interpersonal difficulties in non-western ones [54]. Many studies from western countries support the conceptualization of NSSI as a risk factor for future SI and SB in patients with DD and other psychiatric issues [15,16,20]. Similarly, a strong relationship between NSSI and SB emerged in several studies from non-western countries [54]. Thus, the presence of the NSSI phenomenon in the clinical picture of depressive and other psychiatric disorders could represent a “transcultural” negative prognostic factor in terms of suicide risk. Our findings are consistent with this view, given that, in a sample of adolescents suffering from depression, those who self-injured had significantly higher SI rates than non-self-injuring ones. That is why we argue that it could be useful to highlight the NSSI engagement when diagnostically defining a DD, similarly to what occurred with the newly introduced “with anxious distress” specifier for MDD in DSM-5 [55]. DSM-5 specifiers allow defining subgroups of patients with a disorder who share specific characteristics relevant for the best management of the disorder they apply to [56]. The advantage of the new specifier “with anxious distress” was to routinely record information about anxiety in patients with MDD, given that anxiety was found to predict suicide risk [55]. From our point of view, also NSSI seems to apply for a potential role as a “specifier” of DDs, given its potentially negative prognostic value and relevance in terms of therapeutic management. Notably, in the case of NSSI engagement, not only the choice of the pharmacological treatment could be crucial, given the heightened suicide risk, but also the type of psychotherapeutic intervention is likely to be very important. In fact, at least in cultures where the endorsement of NSSI is associated with high levels of emotional dysregulation, as clearly emerged from our sample, treatments emphasizing emotion regulation as a core component (e.g., dialectical behavior therapy, emotion regulation group therapy) could be particularly useful also in improving the symptomatology of the DD by reducing the self-injury component [57].

Further large-scale longitudinal studies are needed to support the hypothesis that NSSI may be a specifier of DDs, clarifying the dynamic relationship between the psychopathology of self-harm and depression and the potential NSSI role as a predictor of treatment outcome and prognostic indicator in DDs.

Strengths and limitations of the study. The main strength of this study was that the sample was very homogeneous because accounting for adolescent individuals all diagnosed with a depressive disorder according to DSM-5 criteria. This study also had important limitations that should be highlighted. Firstly, the sample was relatively small, so it is difficult to generalize the data. Second, the cross-sectional design of the study does not allow us to make causal inferences. Furthermore, the directionality of the association between depression and self-injuring could be not clear. Third, we used the dichotomized item 8 of the CDI 2-SR as a marker of suicidal ideation, which is not a robust variable composed of more than one item. Fourth, the high preponderance of females in the “DD + NSSI” group could be a confounding factor in the presence of higher suicidal ideation and depression compared with the “DD” group, given that females had themselves higher levels of suicidal ideation and more depressive symptoms than males. Due to the small size of the present sample, we could not perform distinct analysis splitting on sex. Further larger-scale studies would aim to evaluate the impact of NSSI engagement on the clinical picture of adolescent depression controlling for sex.

## 5. Conclusions

Depression represents a well-defined clinical entity, showing a dramatically increasing prevalence during the COVID-19 emergency. NSSI is a clinical entity of still uncertain definition, the subject of numerous studies, and increasing in clinical practice. Depression and NSSI frequently co-occur, mainly among adolescents.

In our sample of adolescents suffering from depression, individuals engaging NSSI were mostly females, had higher rates of SI, were significantly more emotionally dysregulated, depressed, and anxious than those not endorsing NSSI. In this way, NSSI seemed to characterize more severe clinical pictures of adolescent depression, applying for a potential role as a “specifier” of DDs. Further large-scale longitudinal studies are needed to support this hypothesis, clarifying the dynamic relationship between the psychopathology of self-harm and depression and the potential NSSI role as a predictor of treatment outcome in DDs. This field of research could represent critical opportunities for prevention and therapeutic interventions.

## Figures and Tables

**Table 1:** Descriptive statistics of demographic and clinical data.

**Demographics**	
Total sample	56
“DD + NSSI” group	31 (55.4%; F:M ratio 5:1)
“DD” group	25 (44.6%; F:M ratio 1:1)
Age (years)	14.5 ± 1.8
Sex (females)	39 (69.6%)
Sex (males)	17 (30.1%)
**Type of Depressive Disorder**	
Major depressive disorder	45 (80.3%)
Persistent depressive disorder with major depressive episodes	5 (8.9%)
Persistent depressive disorder with pure dysthymic syndrome	3 (5.4%)
Unspecified depressive disorder	1 (1.8%)
Other specified depressive disorder	1 (1.8%)
Disruptive mood dysregulation disorder	1 (1.8%)
**Psychopharmacological Treatment**	
Antidepressants	16 (28.6%)
Mood stabilizers	10 (17.9%)
Antipsychotics	5 (8.9%)
Combined	6 (10.7%)
**Clinical Assessment**	
CDI 2-SR	69.7 ± 13.3
CDI 2-SR depressed mood/somatic symptoms	67.7 ± 13.7
CDI 2-SR negative self-esteem	65.2 ± 12.9
CDI 2-SR ineffectiveness	67.5 ± 12.9
CDI 2-SR interpersonal problems	65.2 ± 14.6
CDI 2-PV	70.7 ± 9.4
DERS	105 ± 25.7
Suicidal ideation (yes)	42 (75%; F:M ratio 3.7:1)
SCARED-SR	39.9 ± 17.0
SCARED-PV	30.7 ± 12.4

DD: depressive disorder; NSSI: non-suicidal self-injury; F: female; M: male; CDI 2: Children’s Depression Inventory 2; SR: self-report; PV: parent version; DERS: Difficulties in Emotion Regulation Scale; SCARED: Screen for Child Anxiety-Related Emotional Disorders. Values are reported as number and percentage or mean ± standard deviation. DERS data = 54; SCARED-PV data = 55.

**Table 2:** Between-group statistical comparisons of clinical variables in depressed females versus depressed males.

	Females (39)	Males (17)	Student’s *t*-test/M-W-U-test		
Variables	M ± SD or *n* (%)	M ± SD or *n* (%)	*p*-Value	Statistic	Cohen’s d
CDI 2-SR	71.3 ± 13.3	66.2 ± 12.9	0.1854	t = 1.3413	0.3898
CDI 2-SR depressed mood/somatic symptoms	68.5 ± 13.1	66.1 ± 15.1	0.5504	t = 0.6010	0.1747
CDI 2-SR negative self-esteem	67.9 ± 11.8	59.2 ± 13.8	0.0279 *	W = 455.0	NA
CDI 2-SR ineffectiveness	69.4 ± 13.6	63.1 ± 10	0.0886	t = 1.7342	0.5040
CDI 2-SR interpersonal problems	65.7 ± 14.4	64 ± 15.5	0.6850	t = 0.4079	0.1185
CDI 2-PV	70.1 ± 10.3	72 ± 7.1	0.5047	t = 0.6716	−0.1952
DERS	108.5 ± 24.7	96 ± 26.8	0.1102	t = 1.6252	0.4938
Suicidal ideation (yes)	33 (84.6%)	9 (52.9%)	0.0163 *	NA	NA
SCARED-SR	45.5 ± 15.6	26.9 ± 13	0.0001 *	W = 544.5	NA
SCARED-PV	31.2 ± 12.7	29.7 ± 12.1	0.6923	t = 0.3979	0.1161

M-W-U-test: Mann–Whitney U-test; M: mean; SD: standard deviation. DERS data = 15 for the males group; SCARED-PV data = 38 for the females group. W: M-W-U-test. t: Student’s *t*-test. * *p* < 0.05.

**Table 3:** Between-group statistical comparisons of clinical variables in patients suffering from depression who endorse NSSI (“DD + NSSI” group) versus patients who do not endorse NSSI (“DD” group).

	“DD + NSSI” Group (31)	“DD” Group (25)	Student’s/Welch’s *t*-test/MWU test		
Variables	M ± SD or *n* (%)	M ± SD or *n* (%)	*p*-Value	Statistic	Cohen’s d
CDI 2-SR	73.3 ± 14.0	65.3 ± 11.0	0.0138 *	W = 537.0	NA
CDI 2-SR depressed mood/somatic symptoms	70.6 ± 14.4	64.2 ± 12.1	0.0826	t = 1.7689	0.4755
CDI 2-SR negative self-esteem	70.7 ± 12.2	58.4 ± 10.5	0.0002 *	t = 3.9996	1.0751
CDI 2-SR ineffectiveness	69.4 ± 14.5	65.1 ± 10.4	0.2097	t = 1.2695	0.3413
CDI 2-SR interpersonal problems	68.8 ± 15.1	60.8 ± 12.8	0.0387 *	W = 513.0	NA
CDI 2-PV	70.6 ± 11.5	70.8 ± 6.0	0.9250	t = −0.0947	−0.0239
DERS	113.5 ± 21.2	94.4 ± 27.2	0.0092 *	W = 510.0	NA
Suicidal ideation (yes)	28 (90.3%)	14 (56%)	0.0039 *	NA	NA
SCARED-SR	44.3 ± 17.8	34.4 ± 14.6	0.0153 *	W = 535.0	NA
SCARED-PV	30.4 ± 13.2	31.1 ± 11.6	0.8292	t = −0.2168	−0.0589

DERS data = 30 for the “DD + NSSI” group; 24 for the “DD” group; SCARED-PV data = 24 for the “DD” group. t: Student’s *t*-test except for CDI 2 PV (Welch’s *t*-test). * *p* < 0.05.

## Data Availability

The data presented in this study are available within the article.
